# Links between parasitism, energy reserves and fecundity of European anchovy, *Engraulis encrasicolus*, in the northwestern Mediterranean Sea

**DOI:** 10.1093/conphys/cov069

**Published:** 2016-01-22

**Authors:** Dolors Ferrer-Maza, Josep Lloret, Marta Muñoz, Elisabeth Faliex, Sílvia Vila, Pierre Sasal

**Affiliations:** 1 Department of Environmental Sciences, University of Girona, Girona E-17071, Spain; 2 Centre de Formation et de Recherche sur les Environnements Méditerranéens, University of Perpignan Via Domitia, UMR 5110, Perpignan F-66860, France; 3 Laboratoire d’Excellence Corail, CRIOBE, USR 3278 – CNRS – EPHE, CBETM – Université de Perpignan & BP 1013 – 98729, Papetoai, Moorea, French Polynesia

**Keywords:** *Engraulis encrasicolus*, fecundity, fish condition, parasitism

## Abstract

The European anchovy, *Engraulis encrasicolus* L. 1758, is one of the most sought-after target species in the northwestern Mediterranean Sea. However, this stock currently consists of small individuals, and landings are reported to have decreased considerably. The main purpose of this study was to assess, for the first time, the interrelationships between size, fecundity, energy reserves and parasitism in female anchovies, in order to analyse the potential implications for the health of northwestern Mediterranean anchovy stocks arising from the current shortage of large individuals. Results revealed that smaller individuals show lower fecundity, lower lipid content and a higher intensity of certain parasites. As it is known that smaller individuals now predominate in the population, the relationships found in this study indicate that the health of anchovies from the northwestern Mediterranean is currently impaired.

## Abstract (precis)

This study assesses, for the first time, the interrelationships between size, fecundity, energy reserves and parasitism in female European anchovy, in order to analyse the potential implications for the health of the northwestern Mediterranean anchovy stock arising from the current shortage of large individuals.

## Introduction

The European anchovy, *Engraulis encrasicolus* L. 1758, is a small planktivorous pelagic fish with a wide distribution range comprising the Atlantic coast of Europe and western Africa, the Mediterranean Sea and the Black Sea (Fischer *et al.*, 1987). The global catch of European anchovy in 2012 was 489,297 tonnes, which is 20% less than in 2003 (Food and Agriculture Organization of the United Nations, 2014). In the western Mediterranean Sea, anchovy remains one of the most sought-after target species by commercial trawlers or purse seiners, although landings of this species have been steadily declining since 2000 (Palomera *et al.*, 2007). Indeed, although the abundance of anchovy in the northwestern Mediterranean Sea remains relatively high, its biomass and the mean size of individuals have diminished dramatically (Van Beveren *et al.*, 2014).

Different hypotheses have been proposed to explain the decrease in anchovy landings. It has been pointed out that the extension of the fishing season has led to recruitment overfishing, which may be affecting the northwestern Mediterranean anchovy stock (Lleonart and Maynou, 2003). Another factor is that the size at first maturity for anchovy is greater than the minimal legal landing size established for this species in the Mediterranean Sea (Palomera *et al.*, 2003; Tsikliras and Stergiou, 2014), and this may be affecting its reproductive potential. In addition, changes in environmental conditions, such as decreasing river flows and increasing water temperatures, have also been identified as possible factors in the decline in anchovy landings (Lloret *et al.*, 2004; Martín *et al.*, 2012). Recently, special attention has been paid to the changes in the size distribution and condition of anchovy and their potential links with its presently low biomass in the northwestern Mediterranean Sea (as recorded by Van Beveren *et al.*, 2014).

Certainly, fish condition parameters are essential for estimating the health and productivity of exploited populations. In particular, they are a highly significant indicator of their condition (reviewed by Lloret *et al*., 2012, 2014). Therefore, studying the energy reserves of anchovies and the lipid storage dynamics throughout their reproductive cycle could shed some light on the status of anchovy stocks in the northwestern Mediterranean Sea. In contrast, parasitism has also been identified as a factor affecting the condition and reproduction of several fish species (e.g. Barber and Svensson 2003; Bagamian *et al.*, 2004; Bean and Bonner 2009). However, only a few studies have analysed the relationships between parasitism, energy reserves and reproduction in commercially exploited fish species in the Mediterranean (Ferrer-Maza *et al.*, 2015, 2014). There is, to our knowledge, only one other study concerning the European anchovy, carried out in the Black Sea, which has looked into the effects of parasites (in this case, nematodes) on the lipid composition of the European anchovy (Shchepkina, 1985). Nevertheless, given that there are a number of well-known cases of collapse in anchovy fisheries and considering the social and economic upheaval such collapses can cause (Pita *et al.*, 2014), the health of anchovy stocks is well worth studying.

Consequently, the aim of the present study was to evaluate the health of the northwestern Mediterranean anchovy stock based on three indicators (energy reserves, fecundity and parasitism) and to discuss the possible implications of the shortage of large individuals in this stock. We analysed the interrelationships among size, fecundity and two important health indicators, namely lipid content and metazoan parasitism, in female anchovies throughout their reproductive cycle. These interrelationships are discussed from biological and ecological perspectives in order to provide information that can be used to improve the management of this stock, which is highly valued but in a poor state.

## Materials and methods

### Fish sampling

**Figure 1:**
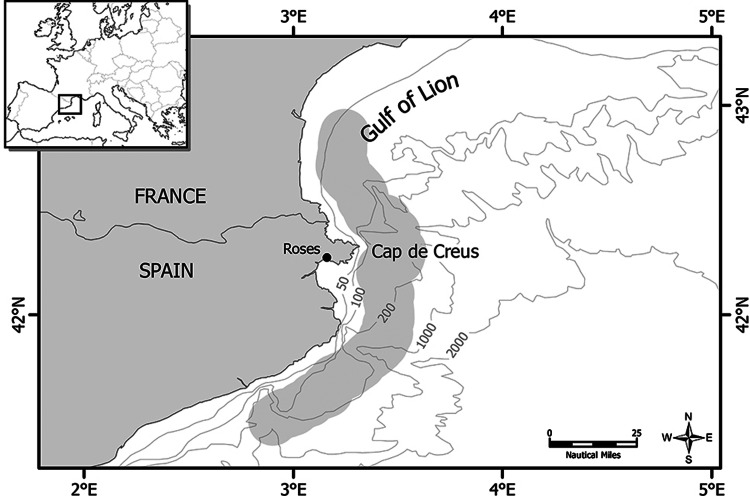
Map of the Cap de Creus (northwestern Mediterranean Sea), showing the port of Roses, where anchovies were sampled. The shaded area represents approximately the zone of capture.

A total of 271 female specimens of European anchovy, *E. encrasicolus*, were collected on a monthly basis from January 2011 to November 2012. The samples were obtained from commercial trawlers and purse seiners at the port of Roses, which is one of the most important commercial fishing harbours in the region (Fig. 1). The specimens were caught near Cap de Creus (northwestern Mediterranean Sea) and its adjacent waters (no further than 25 miles from the shoreline between 42°33′–41°40′N and 3°03′–2°48′E; Fig. 1). The specimens were transported on ice to the laboratory, where they were immediately dissected. For each individual, the total body length (*L*_T_; ±0.1 cm), total body mass (*M*_T_; ±0.01 g), eviscerated body mass (*M*_E_; ±0.01 g), muscle mass (*M*_M_; ±0.01 g) and gonad mass (*M*_G_; ±0.1 mg) were recorded. All the muscle tissue of each specimen was removed and frozen at −20°C for a subsequent determination of lipid content, whereas the ovaries were fixed in 4% buffered formaldehyde for histological processing and estimation of fecundity. The total lengths (*L*_T_) of all specimens ranged from 10.4 to 16.7 cm (mean ± SD = 13.8 ± 1.2 cm, *n* = 271); among these were nine immature specimens ranging from 10.4 to 12.7 cm (11.43 ± 0.63 cm, *n* = 9).

### Classification of reproductive phase

The gonadosomatic index (*I*_G_), which is the relationship between gonad mass (*M*_G_) and eviscerated body mass (*M*_E_) of the females, was calculated as *I*_G_ = 100*M*_G_ *M*_E_^−1^. Subsequently, one ovarian lobe from each specimen was fixed and sliced transversely in its midsection. The resulting slices were embedded in paraffin, cut into 8–10 µm sections, and stained with both Haematoxylin and Eosin and Mallory’s trichrome stains. The latter staining method highlights the zona radiata and its continuity and facilitates the detection of atretic oocytes (Muñoz *et al.*, 2010), i.e. degenerating oocytes that will not be spawned. In the present study we estimated the α atresia, which is the earliest stage of atresia, in which yolked oocytes undergo resorption of yolk. The prevalence of atresia (*P*_A_) was calculated as the proportion of females with observed α-atretic oocytes; the relative intensity of atresia (*I*_A_) was calculated for each specimen as the number of α-atretic oocytes divided by the total number of vitellogenic oocytes (atretic and normal). Following Brown-Peterson *et al*. (2011), all the specimens were classified into six ovarian developmental phases depending upon the presence of specific histological markers. These phases (and the percentage of specimens in each phase) are as follows: immature [3.3%; fish have not reached the sexual maturity (never spawned)]; regenerating (19.9%; sexually mature but reproductively inactive); developing (3.3%; fish with gametes that are beginning to develop); spawning capable (25.5%; advanced, developed gametes ready for the spawning season); actively spawning (30.6%; oocytes in migratory nucleus stage, hydration or ovulation); and regressing (17.4%; massive atresia, which indicates the end of the reproductive cycle).

### Estimation of fecundity

The fecundity estimation was performed on all the specimens that had oocytes in the migratory nucleus stage but did not have recently spawned follicles (*n* = 31). This estimation was performed following the oocyte size–frequency method described by Hunter *et al*. (1985). The females selected were from the actively spawning group and had oocytes in the migratory nucleus stage, i.e. the most advanced stage before hydration and release. All the 31 females were caught between June and August. To ensure that these specimens had not recently released some of their mature oocytes, we used the postovulatory follicle (POF) degeneration key proposed by Alday *et al*. (2010). This key is based on histological features and proposes seven stages of degeneration of POF, as follows: stage I, new POFs; stage II, first signs of degeneration of POFs; stage III, POFs become shrunken; stage IV, clear decrease in size of POFs; stage V, rupture of the cell walls; stage VI, very reduced POFs; and stage VII, no cellular differentiation can be observed. In the present study, any females with recently spawned follicles (stages I–IV) were not selected. Subsequently, slices from the central area of the 31 selected ovaries were weighed (±0.1 mg) and the oocytes separated using a washing process, as described by Lowerre-Barbieri and Barbieri (1993), and sorted by size through several sieves (from 200 to 600 µm). The oocytes were counted and their diameters measured using a computer-aided image analysis system (Image-Pro^®^ Plus 5.1; Media Cybernetics, Inc., Bethesda, MD, USA). Given that the oocyte size distribution followed a two-component mixture model, we applied an algorithm of the mixtools package (Benaglia *et al.*, 2009) for R software (www.r-project.org). This statistical procedure, which had been successfully used in a previous study (Ferrer-Maza *et al.*, 2014), was used to describe quantitatively the properties of the overlapping mixtures, i.e. the two different statistical components of the oocyte size distributions, and to calculate the number of oocytes belonging to the next batch. Given that *E. encrasicolus* is a multiple spawner with indeterminate fecundity (Ganias *et al.*, 2014), the reproductive capacity of the individuals was estimated according to the batch fecundity (*F*_B_), defined as the number of eggs spawned per batch, and the relative batch fecundity (*F*_Brel_), defined as the value of batch fecundity per gram of eviscerated female body mass.

### Determination of energy reserves

To assess the energy reserves of anchovies, first, a visual assessment of their mesenteric fat was carried out following van der Lingen and Hutchings (2005). All the specimens were allocated to one of the five mesenteric fat stages, defined as follows: stage 1, no fat string associated with intestine; stage 2, fat string visible, with thickness less than that of intestine; stage 3, fat string thickness approximately equal to intestinal width; stage 4, fat string thickness greater than intestine, but intestine partly visible; and stage 5, intestine completely covered by fat string. Thereafter, a subsample of muscles (*n* = 210) was selected to perform the lipid extraction and evaluation. In this subsample, all the parasitized specimens and 60% of the non-parasitized specimens were selected in order to have representatives of individuals in different ovarian developmental phases and with different sizes. The total lipid content (percentage wet mass) in muscle was determined following the Soxhlet method described by Shahidi (2001). A lipid musculosomatic index (*I*_LM_) was calculated as *I*_LM_ = 100*ABS*_M_ *M*_E_^−1^, where *ABS*_M_ is the absolute lipid content in muscle, which was obtained by multiplying the lipid content (percentage wet mass) by the wet mass of the muscle. The *I*_LM_ and the mesenteric fat stage were considered as indicators of anchovy condition because the muscle tissue and the mesenterial fat constitute the primary and secondary lipid repositories, respectively, in anchovy (Melo, 1992).

### Evaluation of parasitism

All the specimens were examined for metazoan parasites prior to the removal of muscle and gonads for determination of lipid content and histology. The entire viscera were removed from the body cavity and the internal organs examined using a stereomicroscope. A large subsample of individuals (the first 75 samples) was also examined for metazoan parasites in the musculature. The samples of muscle were examined after filleting and flattening the tissue onto a trans-illumination platform. As no metazoan parasites were found in these examinations, it was decided to regard the number of parasites in the musculature as negligible. When found, the parasites were collected and washed with a saline solution (0.8% NaCl). They were first observed alive and then fixed in permanent preparations. Nematodes were preserved in 70% ethanol and cleared in Amann’s lactophenol, whereas digeneans were fixed in Bouin’s solution under slight coverslip pressure and then stained with Grenacher’s alcoholic borax carmine solution and mounted in Canada balsam. Parasites were morphologically identified to the lowest possible taxonomic level following the available keys and descriptions, such as Petter and Maillard (1988) and Moravec (2007) for nematodes or Gibson *et al.* (2002), Jones *et al*. (2005) and Bray *et al*. (2008) for digeneans. As the identification of the parasite species is normally based on adult features, some of the parasite larvae found in this study could not be identified to the species level. Therefore, the real number of different species might be higher than reported, because there may be several different species within the groups classified as Hemiuridae metacercariae, Didymozoidae metacercariae, *Anisakis* sp. (type I larvae), *Spinitectus* sp., unidentified nematode larvae and Tetraphyllidean plerocercoids. However, although this may be important in certain types of biodiversity studies, in our case, we have assumed that each group we have classified consists of parasites with very similar life cycles and morphology and that the members of each group probably have the same consequences on the condition of their hosts. Following Bush *et al*. (1997), the prevalence of parasites (*P*_P_) was calculated as the proportion of fish infected with a given parasite species, and the individual intensity of the infection was calculated as the number of individuals of a particular species in a single infected host. The mean intensity was calculated as the average number of parasites of a given species found in the infected hosts. Although mean intensity is useful to compare results with other studies because it is one of the most commonly reported parameters, median intensity and its confidence interval (CI) can be a more precise and informative parameter because the majority of parasites are usually found in a very few hosts (Rózsa *et al.*, 2000). Therefore, in the present study the median intensity and its 95% CI were also calculated.

### Data analysis

The aggregated distribution of parasites leads to the concentration of a high proportion of individuals of a particular parasite in a few specimens of the host. As argued by Rózsa *et al*. (2000), it is useful to report the CI for the median intensity of infection. For this reason, the median 95% CI was calculated using the free Quantitative Parasitology 3.0 software (Reiczigel and Rózsa, 2005). This software, which was developed to manage the particularly left-biased frequency distribution of parasites, was also used to compare the prevalences (Fisher’s exact test) and the median intensities (Mood’s median test) for each parasite species throughout the different ovarian developmental phases of the hosts. As normality could not be achieved by any method, several non-parametric tests [Mann–Whitney *U*-test (*U*) and Spearman’s rank correlation coefficient (*r*_s_)] were performed to assess the possible relationships between parasitism and energy reserves (*I*_LM_) and/or fecundity (*F*_Brel_). The level of statistical significance adopted was *P* < 0.05, and a false discovery rate approach was used to counteract the problem of multiple comparisons (Benjamini and Hochberg, 1995; Verhoeven *et al.*, 2005).

## Results

### Reproductive cycle

**Figure 2:**
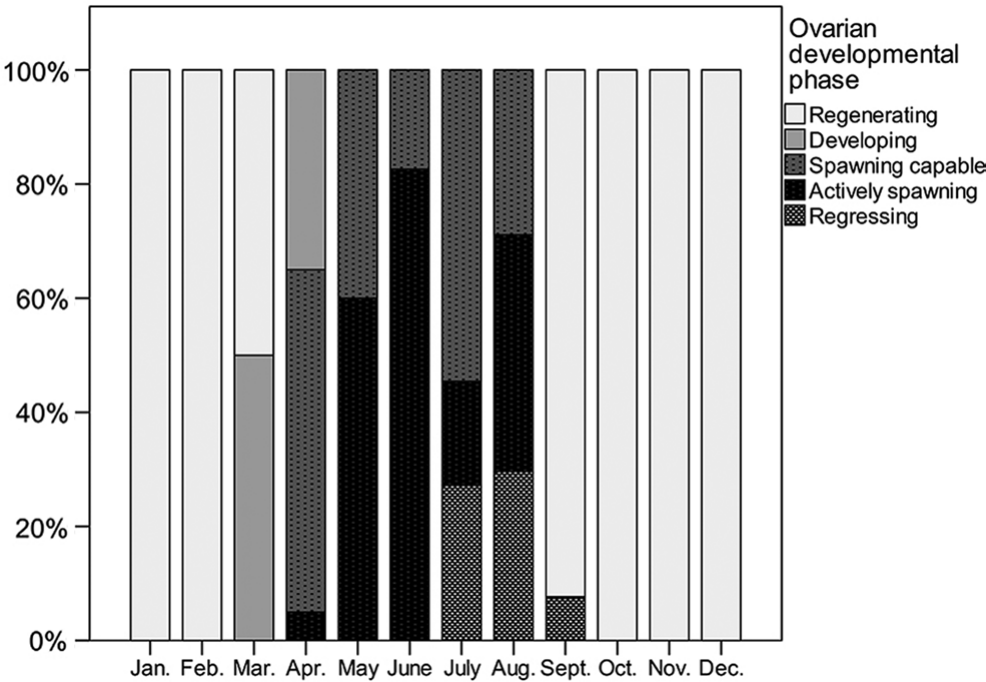
Monthly variations in the relative frequency of ovarian developmental phases in anchovies (from January 2011 to November 2012). Immature specimens are not shown in this figure.

Higher values of *I*_G_ were observed in specimens captured from April until August, with the highest monthly mean in June (mean ± SD = 5.72 ± 2.59, *n* = 23). Indeed, as shown in Fig. 2, the histological examination of the ovaries showed that mature females spent the period from October to February in the regenerating phase until March, when specimens in the developing phase were detected. Between April and August, the spawning period took place, because females in spawning capable or actively spawning phases were observed. Finally, individuals in the regressing phase appeared from July to September. Ovaries with atretic oocytes were not detected among individuals in the regenerating phase, but different intensities of atresia (*I*_A_) were found in specimens during other phases, as follows: (i) developing (*P*_A_ = 22.2%, *I*_A_ = 18.5%); (ii) spawning capable (*P*_A_ = 10.1%, *I*_A_ = 13.7%); and (iii) actively spawning (*P*_A_ = 8.4%, *I*_A_ = 28.2%). Finally, all the individuals in the regressing phase (*P*_A_ = 100%) showed massive atresia (75% < *I*_A_ ≤ 100%).

### Fecundity

**Figure 3:**
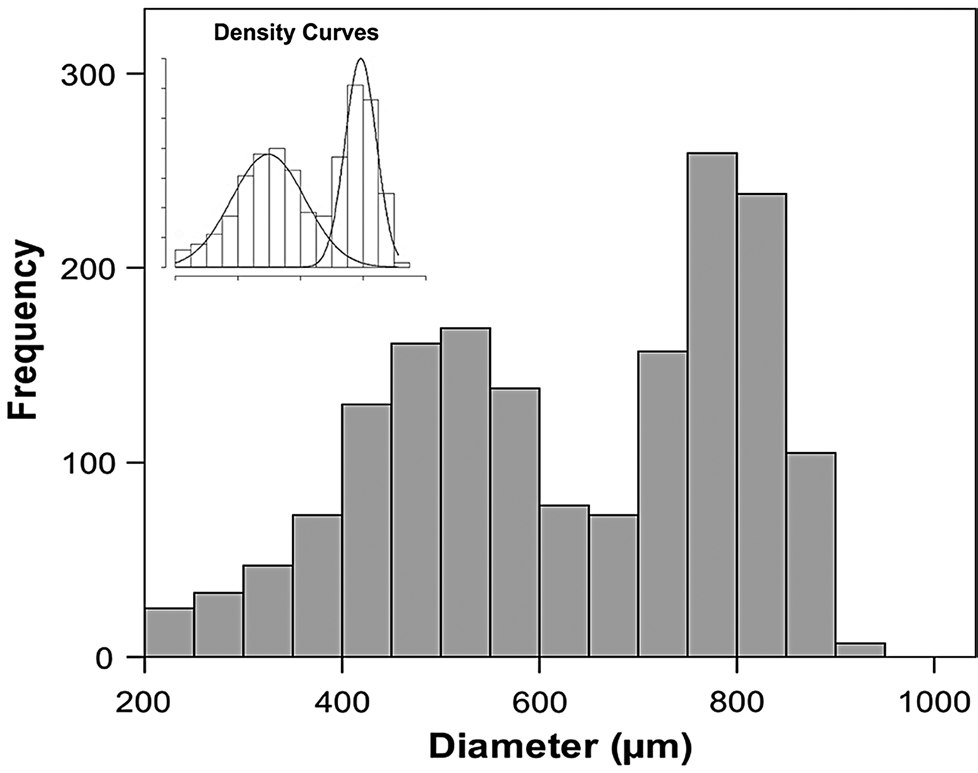
Oocyte diameter–frequency distribution (for oocytes >200 µm), representing most of the female anchovy specimens with oocytes in the migratory nucleus stage. This example corresponds to one female with total body length = 14.9 cm and batch fecundity = 10 672 eggs. The inset shows the two different statistical components of the overlapping mixture distribution. Oocytes with 95% probabilities of belonging to the second component (larger-diameter group) were considered as being part of the next batch.

The batch fecundity (*F*_B_) and the relative batch fecundity (*F*_Brel_) were calculated for 31 females that were in the actively spawning phase and had oocytes at the migratory nucleus stage. The oocyte diameter–frequency distribution of these females showed a bimodal distribution (Fig. 3), with a first component (smaller diameters) containing oocytes at different stages of development (mainly at cortical alveolar and vitellogenic stages) and a second component (larger diameters) containing only oocytes at an advanced stage of maturation (mainly at the migratory nucleus stage), which were considered as being the batch that was about to be released. The *F*_B_ ranged from 981 to 21 750 eggs (11 998 ± 5397, *n* = 31) and was positively related to the size of the specimen. The total length–batch fecundity points fitted a power function regression with the following equation: *F*_B_ = 5.708 × 10^−8^ × *L*_T_^9.640^ (*r*^2^ = 0.66, *n* = 31, *P* < 0.001). The *F*_Brel_ ranged from 96 to 983 eggs (587 ± 227, *n* = 31) and was also positively related to total length as follows: *F*_Brel_ = 1.400 × 10^−5^ × *L*_T_^6.490^ (*r*^2^ = 0.49, *n* = 31, *P* < 0.001).

### Energy reserves

**Figure 4:**
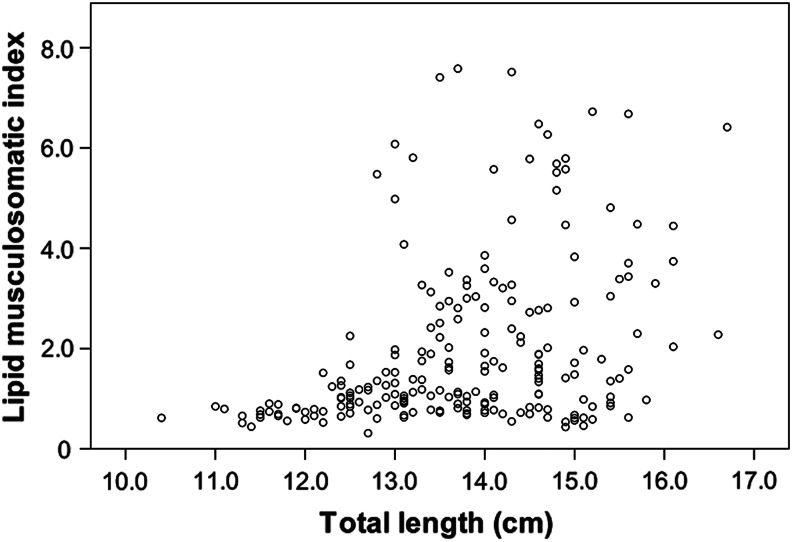
Scatterplot of lipid musculosomatic index (*I*_LM_) of anchovies in relationship to total body length (*n* = 210).

**Figure 5:**
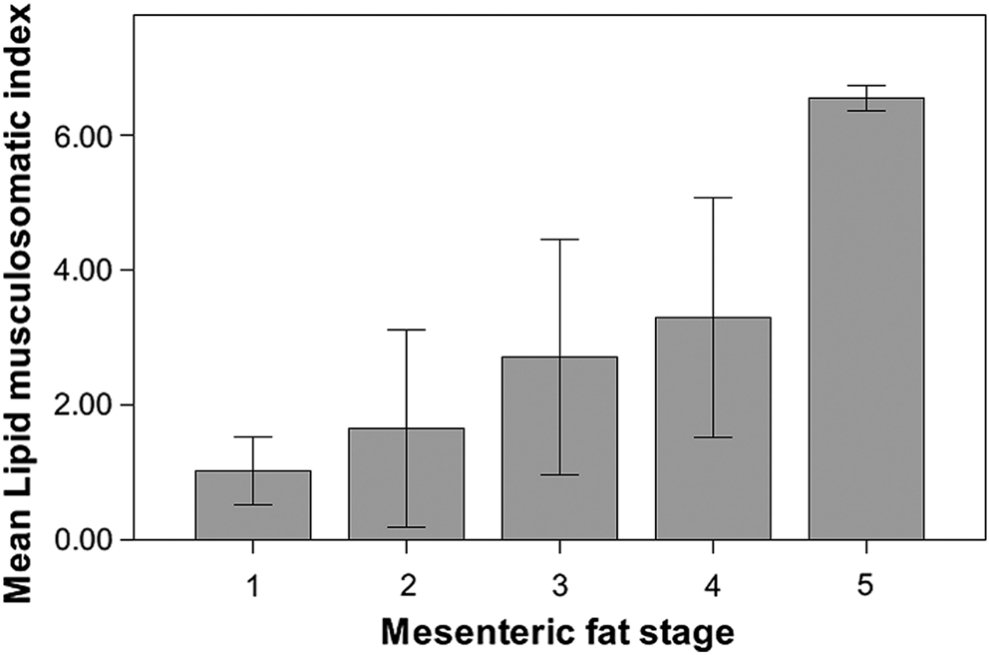
Mean lipid musculosomatic index (*I*_LM_) of anchovies in relationship to mesenteric fat stages. The bars represent ±SD.

**Figure 6:**
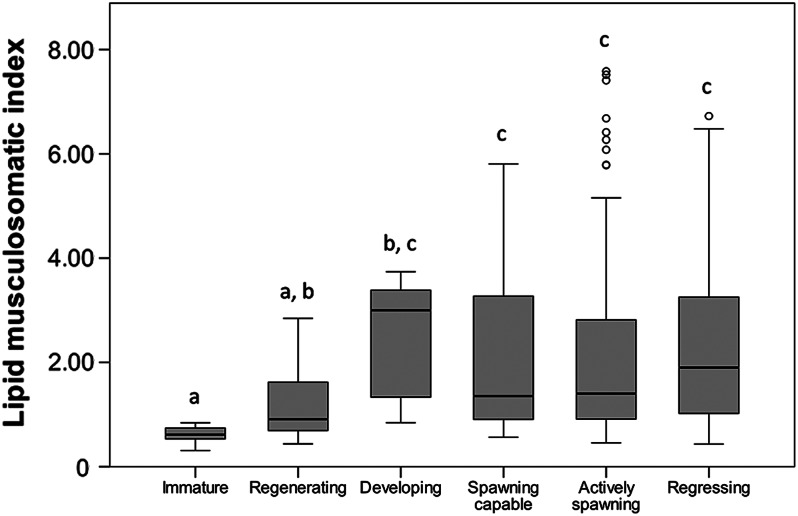
Box-and-whisker plots of lipid musculosomatic index (*I*_LM_) in relationship to the different ovarian developmental phases of anchovies. Significant differences among groups (Kruskal–Wallis test, *P* < 0.05) are indicated with different letters.

In relationship to condition, the lipid musculosomatic index (*I*_LM_) values ranged from 0.31 to 7.58 (1.97 ± 1.65, *n* = 210). The relationship between *I*_LM_ and the total length showed a significant positive correlation (*r*_s_ = 0.38, *n* = 210, *P* < 0.001). Despite the moderate regression coefficient, it can be observed in Fig. 4 that the larger specimens (more than ∼12.5 cm) had a high variability in their *I*_LM_, but all the smaller specimens (up to 12.5 cm) had low values of *I*_LM_. There was also a positive correlation (*r*_s_ = 0.56, *n* = 210, *P* < 0.001) between *I*_LM_ and the mesenteric fat stages. Indeed, as shown in Fig. 5, the mean *I*_LM_ increased as mesenteric fat increased through the five fat stages described in the Materials and methods. In contrast, a Kruskal–Wallis test (*H* = 31.25, *n* = 210, *P* < 0.001) was used to test for differences in the *I*_LM_ between the different ovarian developmental phases (Fig. 6). Immature and regenerating specimens showed lower values of *I*_LM_ than specimens in the other four phases, among which there were no significant differences in their *I*_LM_ values. Finally, there was no relationship between *I*_LM_ and fecundity, because no correlation was found between *I*_LM_ and *F*_B_ (*r*_s_ = 0.13, *n* = 26, *P* = 0.518) or between *I*_LM_ and *F*_Brel_ (*r*_s_ = 0.11, *n* = 26, *P* = 0.591).

### Parasitism

Of all the dissected anchovy specimens, 62.7% were infected with at least one metazoan parasite taxa, with a median intensity that ranged from four to five parasites (95% CI). More than 5000 parasites were classified into eight helminth taxa: three digeneans, four nematodes and one cestode (Table 1). The hemiurid metacercariae (Digenea) were the most prevalent parasites, with a prevalence of 36.16%. However, the parasites with the highest intensity of infection were the tetraphyllidean plerocercoids (Cestoda), with a mean intensity of 197.52 ± 378.57. It should be noted that the only parasite species found at the adult stage, the digenean *Aphanurus stossichi*, was also relatively prevalent (*P*_P_ = 23.62%), as well as the L3 larvae of the nematode *Hysterothylacium aduncum* (*P*_P_ = 18.08%).

**Table 1:** Taxonomic composition, number of infected hosts, prevalence and intensities of metazoan parasites found in the European anchovy, *Engraulis encrasicolus*, from Cap de Creus (northwestern Mediterranean Sea)

Parasite species	Stage	Site	Ovarian developmental phase	Infected hosts	*P*_P_ (*n* = 271)	Intensity		
						Minimum–maximum	Mean ± SD	Median 95% CI
Digenea								
*Aphanurus stossichi* (Monticelli, 1891)	A	S	IM, REG, SC, AS, REGR	64	23.62	(1–25)	3.31 ± 3.50	(2–3)
Hemiuridae	M	P	IM, REG, DEV, SC, AS, REGR	98	36.16	(1–100)	9.79 ± 15.04	(3–4)
Didymozoidae	M	P	DEV, SC, AS	10	3.69	(1–7)	2.60 ± 2.17	(1–6)
Nematoda								
*Anisakis* sp. (type I larvae)	L3	I	REG, SC, AS, REGR	11	4.06	(1–2)	1.09 ± 0.30	(1–1)
*Hysterothylacium aduncum* (Rudolphi, 1802)	L3	I	IM, REG, DEV, SC, AS, REGR	49	18.08	(1–4)	1.45 ± 0.74	(1–1)
*Spinitectus* sp.	L	I	REG, AS	3	1.11	(1–1)	1	1
Unidentified larvae	L	I	REG, SC, AS	21	7.75	(1–3)	1.29 ± 0.64	(1–1)
Cestoda								
Tetraphyllidean	P	I	REG, SC, AS, REGR	23	8.49	(1–1000^a^)	197.52 ± 378.57	(2–100)

The parasite developmental stage, the site of infection and the ovarian developmental phase of hosts are also given. Abbreviations: *n*, sample size; and ’*P*_P_, prevalence. Stage: A, adult; L, immature larvae; L3, third-stage larvae; M, metacercariae; and P, plerocercoid larvae. Preferred site: I, intestines; P, pyloric caeca; and S, stomach. Ovarian developmental phase of hosts: AS, actively spawning; DEV, developing; IM, immature; REG, regenerating; REGR, regressing; and ’SC, spawning capable. ^a^Up to 1000 plerocercoids were counted for four individual hosts, but the real intensities might be higher.

**Figure 7:**
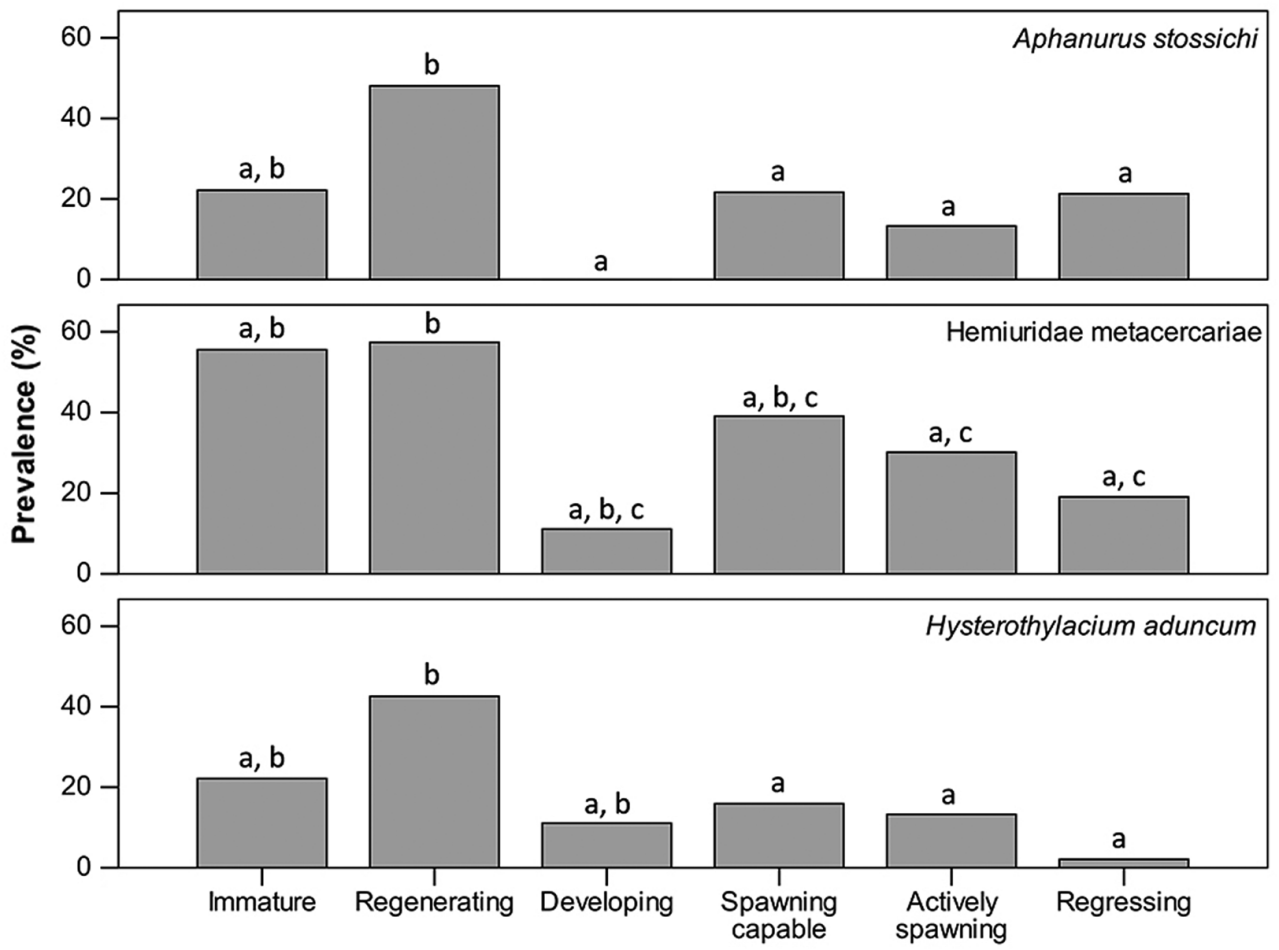
Prevalences (*P*_P_) of parasites in relationship to the different ovarian developmental phases of anchovies. Significant differences among groups (Fisher’s exact test, *P* < 0.05) are indicated with different letters.

In three cases, the digenean *A. stossichi*, the hemiurid metacercariae and the nematode *H. aduncum*, the Fisher’s exact test showed significant differences (*P* < 0.001) between prevalences during the different ovarian developmental phases of their hosts (Fig. 7). Overall, immature anchovy specimens and those in the regenerating phase showed higher parasite prevalences than specimens in the other developmental phases. Despite the significant differences in parasite prevalences, the Mood’s median test showed no difference among the median intensities of any parasite species during the different ovarian developmental phases of their hosts. There was, however, a negative relationship between anchovy length and individual intensity of infection for the aforementioned three parasites, as follows: (i) *A. stossichi*, *r*_s_ = −0.40, *n* = 271, *P* < 0.001, (ii) the hemiurid metacercariae, *r*_s_ = −0.18, *n* = 271, *P* = 0.004; and (iii) *H. aduncum*, *r*_s_ = −0.21, *n* = 271, *P* = 0.002. In contrast, this relationship was positive in the case of the didymozoid metacercariae (*r*_s_ = 0.20, *n* = 271, *P* = 0.002).

### Relationships between parasitism and energy reserves and fecundity

Anchovies in regenerating, spawning capable, actively spawning and regressing phases showed significant differences in their energy reserves (*I*_LM_) and fecundity (*B*_Frel_) related to parasitism (Table 2). Specimens in the regenerating phase and infected by the digenean *A. stossichi* displayed lower median values of *I*_LM_ compared with uninfected specimens (Mann–Whitney *U*-test; Table 2). There was also a negative correlation between individual intensities of infection by this digenean and individual values of *I*_LM_ (Spearman’s rank correlation; Table 2). Similar results were found for the anchovies in spawning capable, actively spawning and regressing phases infected by the hemiurid metacercariae. Similar negative correlations were found for individuals in the actively spawning phase infected by the unidentified nematode larvae as well as for the specimens in the regressing phase infected by *A. stossichi*. Negative relationships were also found between fecundity and infection by *A. stossichi* or *H. aduncum*. Conversely, anchovies infected by the didymozoid metacercariae showed higher median values of *B*_Frel_ than uninfected specimens, and a positive correlation between individual intensities of infection and individual values of *B*_Frel_ was found. Finally, the specimens in the regressing phase infected by *Anisakis* sp. also showed a positive correlation between individual intensities of infection and individual values of *I*_LM_, although no significant differences were found between specimens infected by this nematode and uninfected ones (Mann–Whitney *U*-test). For the remaining parasites (*Spinitectus* sp. and the tetraphyllidean plerocercoids), no significant correlation was found for either the energy reserves or the fecundity of the host, nor was there any significant relationship between parasitism and energy reserves of immature or developing anchovies (whose fecundity cannot be evaluated).

**Table 2:** Results of Mann–Whitney *U*-test used to verify the existence of differences between infected and uninfected *E. encrasicolus* and Spearman’s rank correlation coefficient (*r*_s_) used to evaluate possible relationships between the individual intensities of parasitism and the energy reserves and fecundity of fish

Ovarian developmental phase (*n*)	Parasite	Variable	Mann–Whitney *U*-test						Spearman’s rank correlation		
			*n* Uninf.	*n* Inf.	*Md* Uninf.	*Md* Inf.	*U*	*P*-value^a^	*n*	*r*_s_	*P*-value
Regenerating (54)	*Aphanurus stossichi*	*I*_LM_	23	23	1.090	0.758	175	0.049	46	−0.318	0.031
Spawning capable (69)	Hemiuridae (metacercariae)	*I*_LM_	33	24	2.276	1.044	267	0.037	57	−0.271	0.041
Actively spawning (83)	Hemiuridae (metacercariae)	*I*_LM_	43	22	1.524	1.168	326	0.042	65	−0.262	0.035
	Unidentified nematode larvae	*I*_LM_	54	11	1.547	1.057	177	0.036	65	−0.268	0.031
	*Aphanurus stossichi*	*B*_Frel_	28	3	594.0	416.0	8	0.023	31	−0.419	0.019
	Didymozoidae (metacercariae)	*B*_Frel_	24	7	543.5	701.0	129	0.033	31	0.384	0.033
	*Hysterothylacium aduncum*	*B*_Frel_	28	3	594.0	244.0	10	0.032	31	−0.390	0.030
Regressing (47)	*Aphanurus stossichi*	*I*_LM_	20	10	2.355	1.017	30	0.002	30	−0.585	0.001
	Hemiuridae (metacercariae)	*I*_LM_	23	7	2.035	1.353	40	0.047	30	−0.397	0.030
	*Anisakis* sp.	*I*_LM_	–	–	–	–	–	–	30	0.366	0.047

Abbreviations: Inf., infected fish; *Md*, variable median; *n*, subsample size; and Uninf., uninfected fish. Variables: *B*_Frel_, relative batch fecundity; and *I*_LM_, lipid musculosomatic index. ^a^Asymptotic significances (two-tailed) are displayed for Mann–Whitney *U*-tests with sample size >10 in both groups; otherwise, exact significances [2 × (one-tailed significance)] are given. Only significant results (*P* < 0.05) are presented.

## Discussion

**Figure 8:**
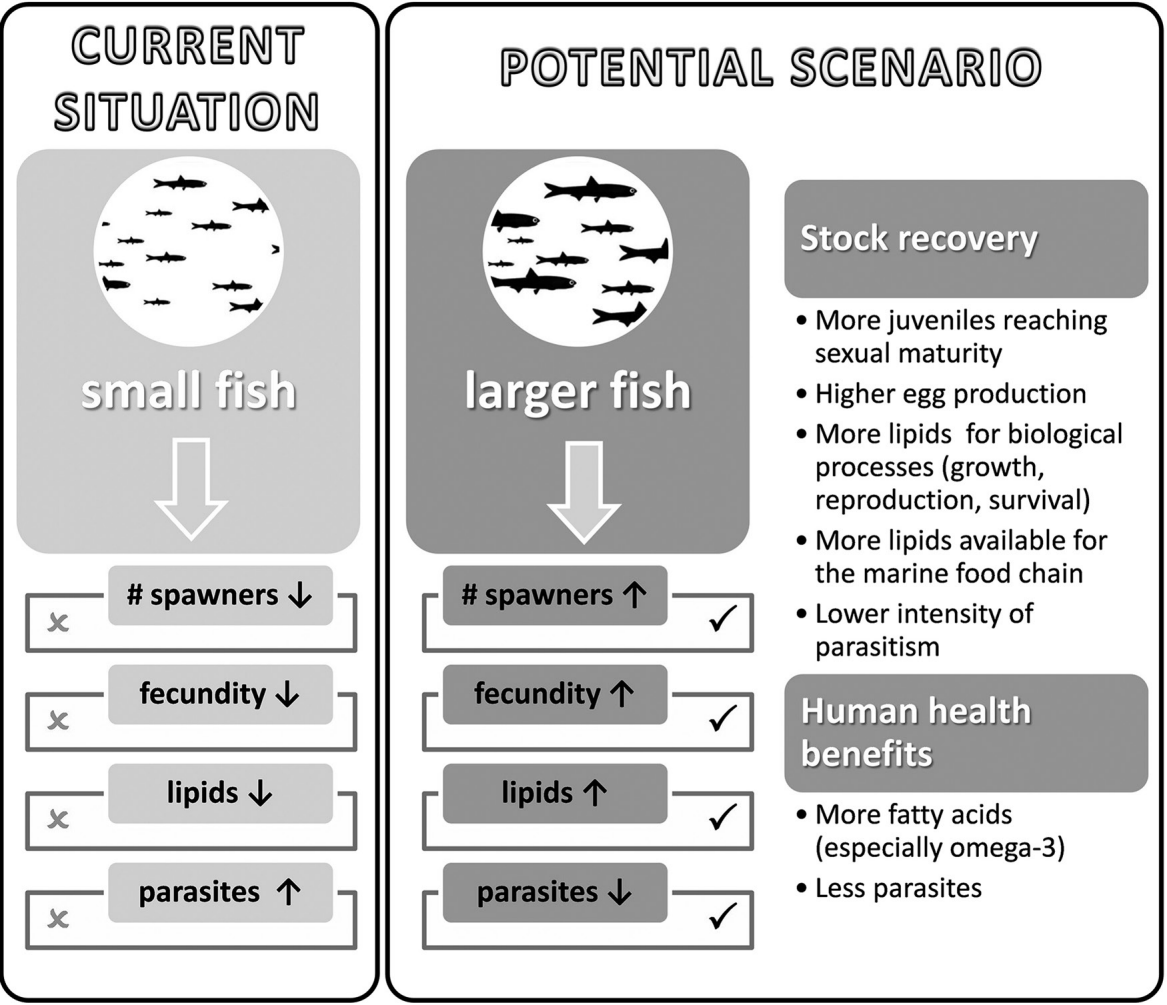
Possible implications for the health of the anchovy stock in a hypothetical scenario in which there is an increase in the median size of anchovy.

Overall, our results revealed that smaller individuals show lower fecundity, lower lipid content and a higher intensity of certain parasites (Fig. 8), which in some cases (e.g. certain digeneans and nematodes) are negatively related to the energy reserves of the host. As it has been shown that smaller individuals now predominate in the population (Van Beveren *et al.*, 2014), the relationships found in the present study might indicate that the fecundity and health status of the anchovies from the northwestern Mediterranean stock are currently impaired.

The results of the present study also show that the spawning season of the European anchovy in the northwestern Mediterranean Sea takes place between April and August, which is consistent with what has been reported by other authors for other regions of the Mediterranean Sea (Tsikliras *et al.*, 2010). The values of fecundity reported in the present study are also coherent with other studies carried out with the European anchovy in the Adriatic Sea (Casavola *et al.*, 1996) or in the northeast Atlantic Ocean (Sanz and Uriarte, 1989; Motos, 1996). The batch fecundity was positively related to total length, which means that larger individuals have a higher reproductive capacity than smaller ones, as is commonly the case with several northwestern Mediterranean commercial fish species (Muñoz *et al.*, 2005; Ferrer-Maza *et al.*, 2014, 2015; Villegas-Hernández *et al.*, 2014, 2015a,b). In the present study, the size of all the immature anchovy specimens we examined exceeded 9 cm, which is currently the minimal landing size for anchovy in the Mediterranean Sea. As suggested by Tsikliras and Stergiou (2014), an increase in the minimal landing size would be more appropriate in order to ensure the recruitment of this species. Indeed, our results suggest that a hypothetical increase in the minimal landing size might not only contribute to allowing juveniles to reach sexual maturity but would also be associated with an increase in the average fecundity of spawners. Furthermore, the absence of a relationship between batch fecundity and energy reserves seems to indicate that the size of anchovy has a greater influence than the condition of the fish on the reproductive potential of this species.

In relationship to energy reserves, the results showed that because the stock currently consists mostly of small individuals (Van Beveren *et al.*, 2014), the energy reserves of individual anchovies and of the whole population are low. We identified two different groups according to the size of the individuals: those larger than 12.5 cm showed strong variations in lipid content in the muscle, whereas all individuals below this size had low energy reserves. These findings are in accordance with the aforementioned work by Van Beveren *et al*. (2014), who produced a comprehensive study using an extensive and reliable data time series (1992–2012) that was compiled from scientific surveys. They found that although the abundance of anchovy had remained relatively high, its biomass and mean size had declined dramatically. According to the authors, the median length for the whole data set was 12.5 cm and the size distribution remained fairly constant from 1992 until 2005, whereupon a sudden shift towards larger individuals was detected. During the following years, a decline in size took place, with the result that the median fish size of the anchovy population of the last 4 years (2009–2012) was smaller than in all preceding years. Furthermore, during this later period, 2009–2012, the analysis of the relative condition factor also indicated that the condition of the anchovies was poor. It seems, therefore, that size and condition are linked and that there is a shift in the energy reserves of anchovies at median lengths of ∼12.5 cm. It should also be noted that these changes in energy reserves are not attributable to the reproductive status of the specimens, because our results showed that there was no depletion of lipids that correlated with the spawning season, i.e. from developing to regressing phases, which is probably because anchovy in the Mediterranean Sea continue feeding during their spawning season (Costalago *et al.*, 2012; Nikolioudakis *et al.*, 2014). Consequently, it can be assumed that, in terms of condition, the state of the stock is better when the median length of the individuals is >12.5 cm because specimens below this size have low energy reserves, which are essential for growing or maintaining fitness during reproduction. Therefore, we may hypothesize that in a potential scenario in which the anchovies can grow to a larger size before they are caught, the overall state of the stock in terms of fecundity, energy reserves and parasitism could improve (Fig. 8).

An accessory result concerning energy reserves is the clear relationship between lipid content in muscle and the mesenteric fat stage using the method proposed by van der Lingen and Hutchings (2005). We therefore consider that the mesenteric fat stage is a good indicator of energy reserves in European anchovy in the northwestern Mediterranean Sea, and we support the incorporation of this cheap, quick and easy method for estimating anchovy energy reserves into other pelagic fish surveys.

Our results on parasitism showed that the metazoan parasite fauna of anchovy in the northwestern Mediterranean is dominated by digeneans and nematodes, most of them in larval stages. Although some parasites (*A. stossichi*, the hemiurid metacercariae and *H. aduncum*) were more prevalent in immature and regenerating specimens, no difference in the median intensity of infection was found during the different ovarian developmental phases. It appears, therefore, that the reproductive status of females during the spawning season does not significantly affect the parasite load. However, another explanation could be that only the healthier individuals are surviving the energy-demanding spawning season. It must be considered that the present study was carried out with wild-caught specimens, and we do not know whether parasitism is effectively causing the death of the individuals with heaviest parasite burdens during the spawning season.

In contrast, individual intensities of parasitism showed that smaller anchovy specimens harboured more parasites (*A. stossichi*, the hemiurid metacercariae and *H. aduncum*) than larger specimens. Conversely, our results also showed that higher intensities of didymozoid metacercariae were found in larger anchovies. There is little research available concerning most of the various parasites that infect anchovy in the Mediterranean Sea, with the exception of nematode infection, on which there is a wide range of research. Indeed, because anisakid nematodes are involved in a disease that affects humans, i.e. anisakidosis, there is a considerable amount of literature concerning infections by these nematodes in anchovy (e.g. Rello *et al*., 2009; Gutiérrez-Galindo *et al*., 2010; De Liberato *et al*., 2013; Serracca *et al*., 2014). Such studies have reported values of prevalence ranging from 0 to 25%, which is in accordance with our results (*H. aduncum P*_P_ = 18.08% and *Anisakis* sp. *P*_P_ = 4.06%). However, one study carried out in the Adriatic Sea (Mladineo *et al.*, 2012) found a 76% prevalence of *Anisakis pegreffii* infection in anchovy, which is much higher than any of the prevalences reported in others parts of the Mediterranean Sea. In contrast to our study, Mladineo *et al*. (2012) reported a positive relationship between anchovy length and the intensity of infection by *Anisakis pegreffii*. However, Rello *et al*. (2009) and De Liberato *et al*. (2013) found no significant correlation between length and intensity of infection. In any case, there appear to be significant differences between different areas of the Mediterranean in the prevalence and intensity of anisakid nematodes, which depends on the abundance of their intermediate and/or definitive hosts (Rello *et al.*, 2009).

The results of the present study indicate that the digenean *A. stossichi*, the hemiurid metacercariae and some unidentified nematode larvae could be affecting the energy reserves of anchovy negatively. This interpretation is reinforced by our results that showed that there was no depletion of lipids that correlated with the spawning season (developing, spawning capable, actively spawning or regressing phases). Therefore, we can hypothesize that the observed differences in the energy reserves could be due to the effect of parasitism rather than that of reproduction. Although most of the relationships were negative, we also found a positive relationship between *Anisakis* sp. and energy reserves, which showed that the impact of parasites is not always evident and straightforward. It should be noted that other biotic and abiotic factors, particularly food availability, can also influence anchovy condition. For example, Brosset *et al.* (2015) demonstrated that the concentration of mesozooplankton has a significant positive influence on anchovy condition in the Gulf of Lion. However, negative effects of nematodes on anchovy energy reserves have also been reported by Shchepkina (1985), who analysed the lipid concentration in the liver and the white and red muscles of anchovy in the Black Sea and found that specimens that were heavily infected by nematodes showed lower lipid concentrations (especially triglycerides) in their tissues than lightly infected specimens.

It appears that *A. stossichi* and *H. aduncum* have a negative relationship with female egg production, whereas the didymozoid metacercariae is positively related. However, these results must be interpreted with caution. It is more likely that these differences in fecundity are attributable to anchovy length rather than parasitism. Indeed, as previously reported, anchovy length is a good indicator of the individual’s fecundity because there is a significant positive relationship between the two variables. Furthermore, when anchovy length vs. individual intensity of infection was analysed, smaller anchovies were found to have more *A. stossichi* and *H. aduncum*, whereas larger anchovies had more didymozoid metacercariae. Therefore, we consider that metazoan parasitism does not significantly affect the fecundity of female anchovies and that the variations we found were because of the influence of body length.

Taken together, our results reveal that the current prevalence of smaller individuals in the northwestern Mediterranean anchovy stock might have several consequences for the health of the stock as a whole, because they have lower fecundity, lower lipid content and higher intensity of certain parasites. We think that the correlations between anchovy size, fecundity, energy reserves and metazoan parasitism found in the present study should be taken into consideration in the management of the European anchovy. Our results can be useful not only because of the economic and ecological implications of further deterioration in anchovy stocks but also for the implications on human health; bigger individuals provide consumers with greater nutritional benefits (more fish oil) and fewer health risks (fewer parasites). In any case, despite the fact that the relationships found in the present study between parasitism, condition and reproduction indicate that there is a link between these parameters, the cause–effect relationships still remain unknown. Therefore, we consider that further research, especially experimental studies that consider other endogenous and exogenous factors that could produce additive, synergistic or antagonistic effects on fish condition and reproduction, should be undertaken to gain a better understanding of the links between fish health and reproduction.

## Funding

This work was supported by the Spanish Ministry of Science and Innovation (research project ref. CTM2009-08602). D.F.-M. benefited from a Formación de Personal Investigador predoctoral fellowship (ref. BES-2010-032618) and J.L. benefited from a ‘*Ramón y Cajal*’ research contract from the Spanish Ministry of Economy and Competitiveness.
